# Cervical and breast cancer screening uptake among women with serious mental illness: a data linkage study

**DOI:** 10.1186/s12885-016-2842-8

**Published:** 2016-10-21

**Authors:** Charlotte Woodhead, Ruth Cunningham, Mark Ashworth, Elizabeth Barley, Robert J. Stewart, Max J. Henderson

**Affiliations:** 1 Institute of Psychiatry, Psychology & Neuroscience, King’s College London, London, UK; 2 Department of Public Health, University of Otago, Wellington, New Zealand; 3 Department of Primary Care and Public Health Sciences, King’s College London, London, UK; 4 Facility of Nursing and Midwifery, King’s College London, London, UK

**Keywords:** Cancer screening, Breast cancer, Cervical cancer, Mammography, Psychoses, Serious mental illness, Data linkage

## Abstract

**Background:**

Breast and cancer screening uptake has been found to be lower among women with serious mental illness (SMI). This study aims to corroborate these findings in the UK and to identify variation in screening uptake by illness/treatment factors, and primary care consultation frequency.

**Methods:**

Linked population-based primary and secondary care data from the London borough of Lambeth (UK) were used to compare breast and cervical screening receipt among linked eligible SMI patients (*n* = 625 and *n* = 1393), to those without SMI known only to primary care (*n* = 106,554 and *n* = 25,385) using logistic regression models adjusted first for socio-demographic factors and second, additionally for primary care consultation frequency.

**Results:**

Eligible SMI patients were less likely to have received breast (adjusted odds ratio (OR) 0.69, 95 % confidence interval (CI), 0.57 - 0.84, *p* < 0.001) or cervical screening (adjusted OR 0.72, CI: 0.60 - 0.85, *p* < 0.001). Schizophrenia diagnosis, depot injectable antipsychotic prescription, and illness severity and risk were associated with the lowest odds of uptake of breast (adjusted ORs 0.46 to 0.59, all *p* < 0.001) and cervical screening (adjusted ORs 0.48 - 0.65, all *p* < 0.001). Adjustments for consultation frequency further reduced effect sizes for all subgroups of SMI patient, in particular for cervical screening.

**Conclusions:**

Women with SMI are less likely to receive breast and cervical cancer screening than comparable women without SMI. Higher primary care consultation rates among SMI patients is likely a mediating factor between SMI status and uptake, particularly for cervical screening - a service organised in primary care. To tackle health disparities linked to SMI, efforts at increasing screening uptake are key and should be targeted at women with other markers of illness severity or risk, beyond SMI status alone.

## Background

People with serious mental illness (SMI), including schizophrenia and bipolar disorder, have higher cancer mortality than others of the same age in the same population without SMI, and there is some evidence that this excess disproportionately affects women [1–3]. Cancer screening reduces cancer mortality; reducing cancer incidence and improving survival [4]. Three recent systematic reviews have found suboptimal cancer screening rates in people with mental disorders. These reviews have included a range of diagnoses, ranging from emotional distress to diagnosed SMI. Where evidence has been disaggregated by type of mental disorder, those with SMI are found to have the lowest odds of screening compared to those without mental ill health [5–7]. Further, the majority of studies reviewed originate from the United States so it is not clear how these results translate into the United Kingdom (UK) context with universal free access to healthcare and organised population-based screening programmes. Absence of cost barriers, and organised screening, have been shown to reduce inequalities in screening coverage, [8, 9] hence more equitable coverage for people with SMI might be expected in the UK.

The Quality and Outcomes Framework (QOF) is a primary care reward and incentive programme which annually records general practice (GP) achievement against clinical and organisational targets and was introduced in 2004 [10]. Targets include measures aimed at improving the physical health care of people with SMI, such as blood pressure monitoring and cervical cancer screening. It is unclear whether such incentivisation results in more widespread uptake of screening.

Barriers to participation in screening programmes among people with experience of mental illness include factors at the service, practitioner, and service user level [11]. Routinely available clinical data sources can be used on a larger scale to investigate specific barriers to screening such as lack of contact with a primary care provider, social deprivation, and factors related to the type and severity of mental illness. The ability to investigate these processes has previously been restricted as data on physical health diagnoses, monitoring and management are mainly recorded in primary care, while detailed classification of SMI diagnosis and mental state is mainly recorded in secondary mental health care records.

This study uses data from a population-based linkage between primary and secondary care records in the London borough of Lambeth (UK) to extend previous knowledge about the uptake of breast and cancer screening in the SMI population; addressing the following questions:

1. Is breast and cervical cancer screening uptake lower for people registered in primary care with SMI compared to those without SMI?
2. Does frequency of contact with primary care explain differences in screening rates between those with and without SMI?
3. Are there psychological factors that predict lower screening uptake within the SMI group?

## Methods

### Setting & data sources

Lambeth is an ethnically diverse borough, with a greater number of Black Caribbean and Black African residents, although fewer South Asian residents than most other areas of London [12] and has high levels of deprivation overall [13]. Pseudonymised primary care data were extracted on 31^st^ October 2013 from the computerised medical records of all except one GP practice (*n* = 48; the missing GP practice had an incompatible IT system) within Lambeth, as part of Lambeth DataNet (LDN). LDN collects demographic data and data on GP consultations, prescriptions, and (QOF) clinical target achievement, as well as clinical information about non-QOF conditions. LDN thus contributed a population of 366,317 registered patients. Secondary care data came from the Clinical Record Interactive Search (CRIS), [14] an application allowing research access to pseudonymised electronic health record data from the South London and the Maudsley NHS Foundation Trust (SLaM). CRIS additionally provides searchable access to de-identified text (unstructured data) from the clinical record, and a range of natural language programming (NLP) applications have been developed to auto-extract structured data from text fields [15].

### Data linkage

CRIS and LDN data were linked and stored by the SLaM Clinical Data Linkage Service (CDLS), which provides a safe haven environment with strict governance arrangements. Data were linked using encrypted NHS numbers, which were subsequently removed and destroyed such that the linked dataset became fully anonymised.

### Measures

#### Primary care data from Lambeth DataNet (LDN)

Data extracted from LDN included 2012/13 QOF-defined SMI status (recorded on the QOF Mental Health register with a diagnosis of schizophrenia, bipolar affective disorder and other psychoses), [10] gender, year of birth, ethnicity, and 2011 defined lower super output area (LSOA). These are geographic areas designed to improve the reporting of small area statistics in England and Wales, and include a mean population of 1500 [16]. Approximate age was calculated by subtracting year of birth from the year of data extraction; information on the LSOA of each patient was used to determine the level of social deprivation in their area using the Index of Multiple Deprivation (IMD-2010) and a conversion from 2001 LSOA to 2011 LSOA values [17]. Frequency of primary care consultation was calculated as the mean number of primary care consultations (including GP, nurse, face-to-face, and telephone) over the three years between October 2010 and 31 October 2013. A binary variable was created to distinguish low (median or below) and high (above median) mean annual number of consultations. Consultation data were coded as missing for two practices which had incorrectly entered data for 2013 GP face-to-face appointments and for numbers entered as negative values. Lastly, data were extracted to identify those who had ever been recorded as having received breast cancer screening (mammography) and cervical cancer screening. The population eligible for mammography was defined as females aged 50 to 70 years inclusive, while that eligible for cervical cancer screening was defined as females aged 25 to 64 years inclusive. To assess adherence to QOF guidelines, those who had received a mammography screen any time in the last three years were identified as recently screened and distinguished from those who had been recorded as being screened outside of the guideline period. Similarly, those who had received a cervical cancer screen any time in the last three years for those aged up to 49 years, or any time in the last five years for those aged 50-64 were identified as recently screened as per recommended guidelines. For both cervical and breast screening, those with a recent screen were coded as 1, while those never screened were coded as 0.

#### Secondary mental health care measures from CRIS

ICD-10 diagnostic codes [18] for any primary or secondary diagnosis of schizophrenia, bipolar affective disorder, and schizoaffective disorder or other non-organic psychoses were extracted (ICD-10 codes F20-29, F25, F31). A binary indicator of higher SMI severity was created which ascertained and coded as 1, any patients with a recorded mental health inpatient stay, treatment under the Mental Health Act, difficulty managing their physical health as recorded in a clinical risk assessment; or contact with Assertive Outreach, Crisis team or A&E liaison team. A separate SMI indicator of ‘risk’ was developed, identifying patients with a history of violent or offending behavior using data from a risk assessment violence and aggression subscale. This coded SMI patients as one if had ever had a recorded history of violence, non-compliance, or a forensic history – and as 0 if none of these issues were recorded. In addition, data were extracted on whether or not the patient had ever been recorded with a prescription of antipsychotics - including a marker of depot injectable medication. Table 1 illustrates a summary of variables, variable descriptions and data sources.

**Table 1:** Variable names, definitions and data sources

Variable	Definition	Data source
SMI status	Recorded on the QOF Mental Health register with a diagnosis of schizophrenia, bipolar affective disorder and other psychoses	LDN
Gender	Male/female	LDN
Age	Date of extraction - date of birth (years)	LDN
Deprivation	Index of Multiple deprivation 2010 score (quintiles)	LDN ONS
Primary care consultation	Mean annual number of GP face-to-face, telephone and home visits and nurse consultations between October 2010–2013	LDN
Breast cancer screening	Read code indicating breast cancer screen (mammography) & date of last screen	LDN
Cervical cancer screening	Read code indicating cervical cancer screen (smear) & date of last screen	LDN
SMI diagnosis	ICD-10 diagnostic code (primary and secondary diagnosis)	CRIS
Depot injectable	Ever recorded as prescribed depot injectable antipsychotic	CRIS
SMI severity	Any record of a mental health inpatient stay, treatment under the Mental Health Act, difficulty managing their physical health as recorded in a clinical risk assessment; or contact with Assertive Outreach, Crisis team or A&E liaison team	CRIS
SMI risk	Ever recorded with a history of violence, non-compliance, or a forensic history (on the risk assessment violence and aggression subscale)	CRIS

Notes. *SMI* Serious Mental Illness, *QOF* Quality and Outcomes Framework, *LDN* Lambeth DataNet, *CRIS* Clinical Record Interactive Search; *ONS* Office for National Statistics, *ICD-10* International Statistical Classification of Diseases (2010)

### Statistical analyses

**Fig. 1:**
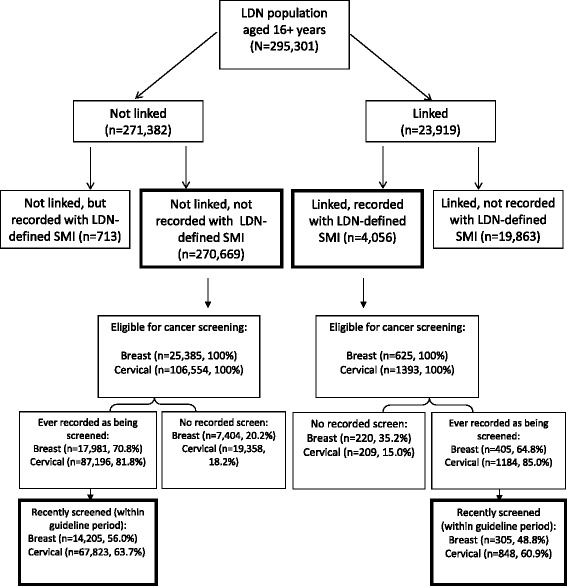
An illustration of Lambeth DataNet (LDN) and the Clinical Record Interactive Search (CRIS) database linkage sample. Also shown is the population eligible for cervical and breast cancer screening, number and proportion of eligible population screened overall and screened within Quality and Outcomes Framework (QOF) guideline periods

Primary care records were used to define SMI status. Those identified in primary care with QOF-defined SMI were compared to those without QOF-defined SMI, who were not linked to secondary care records. Those identified in primary care with SMI but who were not linked, and those who were linked but not recorded in primary care with SMI, were not included in these analyses, as the study aimed to extend prior research by examining SMI characteristics recorded in secondary care associated with screening uptake (see Fig. 1). Descriptive analyses including Pearson’s chi squared tests were used to compare socio-demographic characteristics and consultation frequency between patients with and without SMI patients who were eligible for mammography and/or cervical cancer screening. Separate logistic regression models were run to compare the likelihood of being recorded with a recent breast or cervical screen (versus those never screened) among patients with SMI overall, among patients recorded with specific SMI characteristics and among non-SMI patients. Including non-SMI patients as the comparator in analyses was designed to ease interpretation across analyses. Unadjusted models and models adjusting for socio-demographic characteristics and additionally for primary care consultation frequency were run. Separate analyses were run, but are not presented, to include those who had ever been screened in the numerator (not necessarily within the guideline period) to examine any differences in patterns of association. *P*-values, unadjusted and adjusted odds ratios (OR) and 95 % confidence intervals (CI) are shown. All analyses were conducted using STATA v12 [19].

## Results

### Linkage sample

Overall, data were obtained for LDN patients aged 16 years or over on 31^st^ October 2013 (*N* = 295,301); of these, 8.1 % (*n* = 23,919) were known to secondary mental health care. Among those patients with linked primary and secondary care records, *n* = 4056 (16.9 % of linked sample, 1.37 % of LDN population aged 16+ years overall) were recorded as having SMI by their GP in LDN and were denoted as the SMI group. Overall, 270,669 patients (91.7 % of LDN population) were not recorded with SMI in primary care or linked to secondary care, comprising the group identified without SMI (Fig. 1).

### Study sample

#### Patients eligible for cancer screening

Study sample derivation is illustrated in Fig. 1. We identified 26,010 women in LDN eligible for mammographic screening and 107,947 women eligible for cervical screening, of whom 625 (2.4 %) and 1393 (1.3 %) respectively were recorded in primary care records as having SMI and were also known to secondary mental health services (Fig. 1). Among the eligible populations for breast and cervical cancer screening, SMI status was associated with belonging to an ethnic minority group, greater deprivation, and more frequent primary care consultations. Among those eligible for cervical cancer screening, SMI status was also associated with older age, although this association was not observed for patient eligible for breast screening (Table 2).

**Table 2:** Characteristics of patients eligible for mammography screening for breast and/or cervical screening, by serious mental illness (SMI) status

		Mammography eligible population			Cervical smear eligible population		
		Non-SMI (*n* = 25,385)	SMI (*n* = 625)	p	Non-SMI (*n* = 106,554)	SMI (*n* = 1393)	p
		*n* (%)			*n* (%)		
Age group (years)^a^							
Mammography	Cervical smear			0.709			<0.001
-	25–34	-	-		47449 (44.5)	261 (18.7)	
-	35–44	-	-		27579 (25.9)	382 (27.4)	
50–54	45–54	9035 (35.6)	218 (34.9)		19910 (18.7)	454 (32.6)	
55–64	55–64	11616 (45.8)	296 (47.4)		11616 (10.9)	296 (21.3)	
65–70	-	4734 (18.7)	111 (17.8)		-	-	
Ethnicity				<0.001			<0.001
British/mixed British		7742 (35.8)	189 (33.4)		30953 (33.7)	362 (28.8)	
Irish		528 (2.4)	24 (4.2)		1972 (2.2)	31 (2.5)	
Indian/Bangladeshi/Pakistani		1423 (6.6)	44 (7.8)		5870 (6.4)	75 (6.0)	
Caribbean/mixed Caribbean		3309 (15.3)	142 (25.1)		8880 (9.7)	300 (23.9)	
African/mixed African		2957 (13.7)	71 (12.5)		11382 (12.4)	227 (18.1)	
Chinese/other		991 (4.6)	8 (1.4)		4608 (5.0)	30 (2.4)	
Other white		3741 (17.3)	59 (10.4)		23848 (25.9)	135 (10.7)	
Other black		620 (2.9)	21 (3.7)		2586 (2.8)	73 (5.8)	
Other mixed		307 (1.4)	8 (1.4)		1826 (2.0)	24 (1.9)	
Deprivation quintile				<0.001			<0.001
Most deprived		4706 (19.1)	165 (26.5)		18464 (18.0)	365 (26.4)	
2		5188 (21.1)	123 (19.7)		21223 (20.7)	303 (21.9)	
3		4891 (19.8)	120 (19.3)		21544 (21.0)	272 (19.7)	
4		5024 (20.4)	118 (18.9)		22554 (22.0)	247 (17.9)	
Least deprived		4838 (19.6)	97 (15.6)		18904 (18.4)	197 (14.2)	
Consultations				<0.001			<0.001
Median/below median		7222 (30.8)	64 (10.5)		42642 (44.1)	184 (13.7)	
Above median		16238 (69.2)	545 (89.5)		53982 (55.9)	1158 (86.3)	

^a^Eligible age range for breast screening (mammography) 50–70 years; eligible age range for cervical cancer screening (cervical smear) 25–64 years

### Cancer screening uptake by SMI status

As illustrated in Fig. 1 of the eligible population for cancer screening, *n* = 17,981 (70.8 %) and *n* = 405 (64.8 %) of non-SMI and SMI patients respectively had ever been screened for breast cancer while *n* = 87,196 (65.7 %) and *n* = 1184 (85.0 %) of non-SMI and SMI patients respectively had ever been screened for cervical cancer. The proportion of eligible patients who had received a recent screen was lower among both SMI and non-SMI patients (Fig. 1).

**Table 3:** Associations between serious mental illness (SMI) status and recent receipt of breast and/or cervical screening overall and by SMI characteristic sub-group

	Mammography eligible population (*N* = 26,010)				Cervical smear eligible population (*N* = 107,947)			
	Recorded mammography in last 3 years n (%)	Unadjusted OR (95 % CI)	Adjusted for socio-demographics OR^a^ (95 % CI)	Additionally adjusted for consultation rate OR^b^ (95 % CI)	Recorded cervical smear in last 3/5 years n (%)	Unadjusted OR (95 % CI)	Adjusted for socio-demographics OR^a^ (95 % CI)	Additionally adjusted for consultation rate OR^b^ (95 % CI)
Non-SMI	14205 (65.7)	1.00	1.00	1.00	67823 (77.8)	1.00	1.00	1.00
SMI overall	305 (58.1)	0.72 (0.61 – 0.86)^***^	0.69 (0.57 – 0.84)^***^	0.60 (0.49 – 0.73)^***^	848 (80.2)	1.16 (0.99 – 1.35)	0.72 (0.60 – 0.85)^***^	0.35 (0.29 – 0.42)^***^
SMI by diagnosis								
Schizophrenia	136 (55.1)	0.64 (0.50 – 0.82)^***^	0.59 (0.45 – 0.78)^***^	0.52 (0.40 – 0.69)^***^	270 (76.9)	0.95 (0.74 – 1.22)	0.48 (0.36 – 0.63)^***^	0.24 (0.18 – 0.32)^***^
Bipolar affective disorder	67 (62.0)	0.85 (0.58 – 1.26)	0.89 (0.59 – 1.35)	0.72 (0.47 – 1.10)	231 (87.2)	1.94 (1.35 – 2.78)^***^	1.23 (0.84 – 1.80)	0.50 (0.33 – 0.74)^**^
Other non-organic psychoses	34 (50.8)	0.54 (0.33 – 0.87)^*^	0.53 (0.31 – 0.90)^*^	0.47 (0.27 – 0.80)^**^	153 (74.3)	0.82 (0.60 – 1.13)	0.57 (0.40 –0.80)^**^	0.33 (0.22 –0.47)^***^
Depot injectable								
No	191 (64.3)	0.94 (0.74 – 1.20)	0.97 (0.75 – 1.26)	0.83 (0.64 – 1.09)	524 (82.3)	0.32 (1.08 – 1.62)^**^	0.82 (0.65 – 1.02)	0.36 (0.29 – 0.46)^***^
Yes	80 (49.1)	0.50 (0.37 – 0.68)^***^	0.46 (0.33 – 0.64)^***^	0.39 (0.27 –0.54)^***^	199 (76.3)	0.92 (0.69 – 1.22)	0.48 (0.35 –0.66)^***^	0.26 (0.18 – 0.36)^***^
Any indicator of severity^1^								
No	168 (65.6)	1.00 (0.77 – 1.29)	0.91 (0.69 – 1.21)	0.79 (0.59 –1.06)	411 (82.2)	1.32 (1.05 – 1.66)^*^	0.82 (0.63 – 1.06)	0.40 (0.31 – 0.53)^***^
Yes	137 (50.9)	0.54 (0.43 – 0.69)^***^	0.54 (0.42 –0.70)^***^	0.46 (0.36 – 0.61)^***^	437 (78.5)	1.04 (0.85 – 1.27)	0.65 (0.52 – 0.81)^***^	0.31 (0.25 –0.40)^***^
Any indicator of risk^2^								
No	205 (61.8)	0.84 (0.67 – 1.05)	0.81 (0.63–1.03)	0.70 (0.55 – 0.90)^**^	535 (82.1)	1.31 (1.07 – 1.60)^**^	0.79 (0.63 – 1.00)^*^	0.38 (0.30 –0.49)^***^
Yes	100 (51.8)	0.56 (0.42 – 0.74)^***^	0.53 (0.39 –0.73)^***^	0.46 (0.34 –0.63)^***^	313 (77.3)	0.97 (0.77 – 1.23)	0.62 (0.48 – 0.80)^***^	0.31 (0.24 – 0.41)^***^

Eligible population includes non-linked non-SMI and linked SMI group

*p < 0.05, **p < 0.01, ***p < 0.001

^1^Includes any of: ever had an inpatient stay, any record of being treated under the Mental Health Act, any record of difficulty managing their physical health, or any record of an Assertive Outreach/Crisis/A&E episode

^2^Includes any of: recorded history of violence, recorded history of non-compliance, and any record of a forensic history

^a^Adjusted for age (continuous), ethnicity, and borough-level deprivation

^b^Additionally adjusted for mean annual number of primary consultations

Comparison of screening according to QOF guidelines indicated that SMI patients were less likely to have a record of recent mammography screening compared to those without SMI (Table 3). Adjustments for socio-demographic characteristics, and additionally, for primary care consultation frequency increased the strength of the negative association such that the odds of recent screening were almost 40 % lower among those identified with SMI.

In unadjusted models SMI status was positively associated with recent cervical screening according to guidelines (Table 3). Adjustment for socio-demographic differences between the groups reversed this association - such that being recorded with SMI was associated with reduced odds of recent cervical cancer screening, and adjustment for primary care consultation rate further increased the negative effect size such that the odds of cervical screening were almost 60 % lower among SMI patients.

### Variation in screening receipt by mental illness characteristics

The odds of screening receipt varied across different sub-groups of women with SMI in adjusted models (Table 3). Those with schizophrenia, those ever prescribed depot injectable medication and those ever identified with any indicator of risk or severity, were the least likely to have been screened for breast cancer. Similarly, a diagnosis of schizophrenia and receipt of depot injectable antipsychotic medication were associated with the lowest odds of cervical cancer screening in adjusted models.

## Discussion

After accounting for sociodemographic differences (particularly differences in age), women recorded in primary care as having SMI and also known to secondary mental health services were substantially less likely (with 22–28 % lower odds) to have been screened for breast or cervical cancer than other women in the same population. Unlike previous studies, we were able to explore how SMI characteristics beyond diagnosis were differentially associated with screening, and to explore any potential impact on primary care consultation frequency on screening uptake. For cervical cancer (but not breast cancer) screening uptake, the frequency of primary care contact is a potential mediating factor in the relationship between SMI and screening receipt and so the best estimates of screening frequency and inequality are those in the models adjusted for sociodemographic factors. However, when the higher rates of primary care consultation for women with SMI were taken into account, the difference in cervical screening receipt between women with and without SMI appeared even greater.

The negative effect sizes found in models adjusted for sociodemographic factors were similar for both breast and cervical screening. However, adjusting for frequency of primary care contact had a much greater impact on estimates of cervical than breast screening. This indicates that frequency of primary care contact has a stronger effect on cervical screening rates than mammographic screening rates, as consultation frequency was elevated in the SMI group eligible for both types of screening. This is consistent with differences in the organisation of both national screening programmes, cervical screening being organised within primary care whereas breast screening being organised via a national invitation system. Further, unlike breast screening, cervical screening is incentivised as part of the QOF guidelines for those recorded with SMI. It is unclear whether this has impacted the likelihood of screening uptake, though the adjusted effect size is similar to that reported for women with schizophrenia in a Canadian context without such incentivisation [20]. Nonetheless, breast screening rates were positively associated with frequency of primary care contact suggesting that contact in primary care may promote mammography uptake – perhaps via verbal reminder or encouragement from practitioners. Kodl [21] noted that it is important to take frequency of outpatient care contact into account when assessing screening uptake in people with SMI, as not doing so may obscure differences in screening uptake, although we report reduced screening even without accounting for contact frequency.

Although not presented here, we re-ran analyses to assess the possibility that the difference in screening uptake may be less apparent if the comparison included those ever screened in the numerator. However, the odds associated with SMI status of ever receiving a screen for either cancer remained very similar to analyses including just those with a recent screen, and the pattern by SMI characteristics was also very similar (data available from authors upon request).

In their review of breast and cervical screening among women with a range of mental disorders, Aggarwal et al. [7] identified a need to examine what impact illness severity and treatment have on the relationship between mental illness and screening uptake. Our data linkage allowed us to examine these factors by assessing predictors of screening rates within the SMI group, including diagnosis, receipt of depot medication, and markers of severity. We found that women with schizophrenia had the lowest screening rates, in keeping with other studies that have found that women with more severe mental illness are less likely to be screened [22, 23]. Characterising people with SMI on dimensions other than diagnosis provides a richer understanding of which patients may be most at risk of reduced healthcare including poorer uptake of screening.

Receipt of depot medication can be a more specific indicator of severity of mental illness and engagement with health services. This group had the lowest receipt of screening, which may relate to reluctance to engage with health services leading independently to low screening uptake and the need for depot medication, but may equally relate to difficult engagement with mental health services making people less likely to seek out other care such as cancer screening. Further, those prescribed depot injectable medication may comprise a more unwell group which may hinder uptake of screening for other unmeasured reasons. Other markers of risk and severity were also associated with being less likely to attend screening. Markers of risk and severity were more strongly predictive of uptake of mammographic screening than cervical screening. After adjustment for sociodemographic factors, women without indicators of severity or risk and not on depot medication were not significantly less likely than women without SMI to be screened for breast or cervical cancer. When frequency of primary care visits was taken into account, women without these indicators remained not significantly less likely to receive mammographic screening (with the exception of those with no indicators of risk), but were less likely to receive cervical cancer screening. This difference may be related to mammographic screening being offered at an unfamiliar location with unfamiliar staff, factors which may make attendance more difficult for those with more severe illness.

The finding of a 22–28 % reduction in screening uptake in women with SMI after accounting for demographic factors is in keeping with the international literature [5–7]. For example, a pooled meta-analysis of studies of mammography uptake in the context of mental illness found a 29 % reduction in the odds of mammography in women with mental illness, and a 46 % reduction in women with SMI [5]. However, as noted in the introduction, the majority of studies on which this meta-analysis and other reviews are based were conducted in the United States, where there are no population - based organised screening programmes. In a previous UK study, [22] breast screening registrations were linked to mental health service use and no difference in mammography receipt for women known to mental health services was found overall, but women with a psychosis diagnosis (OR 0.33) or a history of compulsory treatment (OR 0.40) had reduced screening receipt. This study focused on mammography and dates from nearly a decade prior to ours, suggesting that the situation has not changed markedly over this time. Other UK evidence suggests that late diagnosis of cancers may not be a significant factor in poor cancer survival people with SMI, but this evidence comes from a study which examined all cancers combined, and all ages, and so it is not possible to draw conclusions about stage at diagnosis of breast or cervical cancers in screening age populations from these results [24].

### Strengths and limitations

This study is strengthened by the availability of linked primary and secondary care data, which provides information on mental health symptom severity, mental health service receipt, and diagnosis to enable investigation of differences within the group of people with SMI. Further, linkage with population-based primary care records allowed the identification of a direct comparison population. Information held by general practice on cervical screening is likely to be complete. Since mammography is not performed in primary care, information may be less complete, particularly for those who consult less often, although we were able to adjust for consultation frequency.

This study did not include all those who were identified as having SMI in primary care data, but was restricted to those who were also known to secondary mental health services. This group may fare better (because of higher rates of health service contact providing more opportunities for reminders about screening) or worse (because of having more severe illness or more fragmented care) than those not in current contact with secondary services, and so these findings may not apply to the entire group registered with SMI in general practice. However, the decision to compare just those with SMI known to secondary care was made in order to extend currently available knowledge by enabling us to examine the role of illness/secondary mental health care factors not available for those known only to primary care. Whether screening rates are different for SMI patients known only to primary care may be explored in a later study. Lastly, while our findings are representative of a limited geographic area characterised by high levels of deprivation – potentially limiting generalisability – our effect sizes are consistent with those reported in studies internationally, and indicate that screening uptake is reduced for SMI patients even in a setting where screening, primary care and secondary care are provided free at the point of access.

## Conclusions

This study provides up to date information about cancer screening in adults using mental health services in the UK, showing that breast and cervical cancer screening receipt is lower in women with SMI than other women even in the context of free primary care, organised screening and incentives to provide screening, and despite more frequent contact with primary care. It also demonstrates that individual and treatment related factors beyond diagnosis are associated with reduced likelihood of screening. Efforts to improve screening coverage for women with SMI will be important for improving cancer survival for this group. If we are to tackle health disparities linked to SMI status then increasing uptake of cancer screening for women with SMI must be a key element. Our findings indicate the potential benefits of incorporating policies which target efforts at encouraging greater screening uptake among women with other markers of severity or risk, beyond SMI status alone.

## References

[1] Saha S, Chant D, McGrath J (2007). A Systematic Review of Mortality in Schizophrenia: Is the Differential Mortality Gap Worsening Over Time?. Arch Gen Psychiatry.

[2] Bushe CJ, Hodgson R (2010). Schizophrenia and Cancer: In 2010 Do We Understand the Connection?. Can J Psychiatry.

[3] Kisely S, Crowe E, Lawrence D (2013). Cancer-related mortality in people with mental illness. JAMA Psychiatry.

[4] Centres for Disease Control and Prevention (CDC). Cancer Screening Tests.http://www.cdc.gov/cancer/dcpc/prevention/screening.htm. Accessed 17 Oct 2016.

[5] Mitchell AJ, Pereira IES, Yadegarfar M, Pepereke S, Mugadza V, Stubbs B (2014). Breast cancer screening in women with mental illness: comparative meta-analysis of mammography uptake. Br J Psychiatry.

[6] Lord O, Malone D, Mitchell AJ (2010). Receipt of preventive medical care and medical screening for patients with mental illness: a comparative analysis. Gen Hosp Psychiatry.

[7] Aggarwal A, Pandurangi A, Smith W (2013). Disparities in Breast and Cervical Cancer Screening in Women with Mental Illness. Am J Prev Med.

[8] Espinas JA, Aliste L, Fernandez E, Josep MA, Tresserras R, Borras JM (2011). Narrowing the equity gap: The impact of organized versus opportunistic cancer screening in Catalonia (Spain). J Med Screen.

[9] Siminoff LA, Ross L (2005). Access and equity to cancer care in the USA: a review and assessment. Postgrad Med J.

[10] Health and Social Care Information Centre. Quality and Outcomes Framework (QOF). http://content.digital.nhs.uk/qof. Accessed 17 Oct 2016.

[11] Barley EA, Clifton A, Burgess C, Clement S, Ohlsen R, Ramluggun P (2015). Identifying barriers and facilitators to cancer screening uptake by people living with a diagnosis of mental illness to inform policy and practice.

[12] Office of National Statistics. 2011 Census: KS201UK Ethnic group, local authorities in the United Kingdom. http://www.ons.gov.uk/peoplepopulationandcommunity/culturalidentity/ethnicity/datasets/2011censussmallpopulationtablesforenglandandwales; 2011. Accessed 17 Oct 2016.

[13] English Indices of Deprivation 2010. Department of Communities and Local Government. https://www.gov.uk/government/statistics/english-indices-of-deprivation-2010; 2011. Accessed 17 Oct 2016.

[14] Stewart R, Soremekun M, Perera G, Broadbent M, Callard F, Denis M (2009). The South London and Maudsley NHS foundation trust biomedical research centre (SLAM BRC) case register: development and descriptive data. BMC Psychiatry.

[15] Perera G, Broadbent M, Callard F, Chang C-K, Downs J, Dutta R, et al. Cohort profile of the South London and Maudsley NHS Foundation Trust Biomedical Research Centre (SLaM BRC) Case Register: current status and recent enhancement of an Electronic Mental Health Record derived data resource. BMJ Open (in press). 10.1136/bmjopen-2015-008721 PMC4785292 26932138

[16] Office of National Statistics. Super Output Area (SOA). https://neighbourhood.statistics.gov.uk/HTMLDocs/nessgeography/superoutputareasexplained/output-areas-explained.htm; 2015. Accessed 17 Oct 2016.

[17] Indices of Deprivation 2010: IMD10 Scores for LSOAs 2011. Department for Communities and Local Government. http://data.london.gov.uk/dataset/indices-deprivation-2010/resource/7c1858ae-74b2-46d5-a713-731155b87bed; 2011. Accessed 17 Oct 2016.

[18] World Health Organization 2010. International Statistical Classification of Diseases and Related Health Problems - 10th Revision. http://apps.who.int/classifications/icd10/browse/2010/en; Accessed 17 Oct 2016.

[19] StataCorp (2011). Stata Statistical Software: Release 12.

[20] Martens PJ, Chochinov HM, Prior HJ, Fransoo R, Burland E, Need To Know Team (2009). Are cervical cancer screening rates different for women with schizophrenia? A Manitoba population-based study. Schizophr Res.

[21] Kodl MM, Powell AA, Noorbaloochi S, Grill JP, Bangerter AK, Partin MR (2010). Mental health, frequency of healthcare visits, and colorectal cancer screening. Med Care.

[22] Werneke U, Horn O, Maryon-Davis A, Wessely S, Donnan S, McPherson K (2006). Uptake of screening for breast cancer in patients with mental health problems. J Epidemiol Community Health.

[23] Carney CP, Jones LE (2006). The influence of type and severity of mental illness on receipt of screening mammography. J Gen Intern Med.

[24] Chang C-K, Hayes RD, Broadbent MTM, Hotopf M, Davies E, Møller H (2014). A cohort study on mental disorders, stage of cancer at diagnosis and subsequent survival. BMJ Open.

