# Correction to: Cervical and breast cancer screening uptake among women with serious mental illness: a data linkage study

**DOI:** 10.1186/s12885-019-5357-2

**Published:** 2019-02-15

**Authors:** Charlotte Woodhead, Ruth Cunningham, Mark Ashworth, Elizabeth Barley, Robert J. Stewart, Max J. Henderson

**Affiliations:** 1 Institute of Psychiatry, Psychology & Neuroscience, King’s College London, London, UK; 2 Department of Public Health, University of Otago, Wellington, New Zealand; 3 Department of Primary Care and Public Health Sciences, King’s College London, London, UK; 4 Facility of Nursing and Midwifery, King’s, College London, London, UK

**Correction to: BMC Cancer (2016) 16:819**

10.1186/s12885-016-2842-8

Following publication of the original article [1], the authors notified us of an error in the reported percentages in Table 3.

**Table 3:** Associations between serious mental illness (SMI) status and recent receipt of breast and/or cervical screening overall and by SMI characteristic sub-group

	Mammography eligible population (*N*=26,010)				Cervical smear eligible population (*N*=107,947)			
	Recorded mammography in last 3 years n (%)	Unadjusted OR (95% CI)	Adjusted for socio-demographics OR^a^ (95% CI)	Additionally adjusted for consultation rate OR^b^ (95% CI)	Recorded cervical smear in last 3/5 yrs n (%)	Unadjusted OR (95% CI)	Adjusted for socio-demographics OR^a^ (95% CI)	Additionally adjusted for consultation rate OR^b^ (95% CI)
Non-SMI	14205 (56.0)	1.00	1.00	1.00	67823 (63.7)	1.00	1.00	1.00
SMI overall	305 (48.8)	0.72 (0.61 - 0.86)^***^	0.69 (0.57 - 0.84)^***^	0.60 (0.49 - 0.73)^***^	848 (60.9)	1.16 (0.99 - 1.35)	0.72 (0.60 - 0.85)^***^	0.35 (0.29 - 0.42)^***^
SMI by diagnosis								
Schizophrenia	136 (47.9)	0.64 (0.50 - 0.82)^***^	0.59 (0.45 - 0.78)^***^	0.52 (0.40 - 0.69)^***^	270 (57.3)	0.95 (0.74 - 1.22)	0.48 (0.36 - 0.63)^***^	0.24 (0.18 - 0.32)^***^
Bipolar affective disorder	67 (50.4)	0.85 (0.58 - 1.26)	0.89 (0.59 - 1.35)	0.72 (0.47 - 1.10)	231 (67.9)	1.94 (1.35 - 2.78)^***^	1.23 (0.84 - 1.80)	0.50 (0.33 - 0.74)^**^
Other non-organic psychoses	34 (41.5)	0.54 (0.33 - 0.87)^*^	0.53 (0.31 - 0.90)^*^	0.47 (0.27 - 0.80)^**^	153 (56.7)	0.82 (0.60 - 1.13)	0.57 (0.40 - 0.80)^**^	0.33 (0.22 - 0.47)^***^
Depot injectable								
No	191 (53.4)	0.94 (0.74 - 1.20)	0.97 (0.75 - 1.26)	0.83 (0.64 - 1.09)	524 (63.1)	0.32 (1.08 - 1.62)^**^	0.82 (0.65 - 1.02)	0.36 (0.29 - 0.46)^***^
Yes	80 (42.6)	0.50 (0.37 - 0.68)^***^	0.46 (0.33 - 0.64)^***^	0.39 (0.27 - 0.54)^***^	199 (58.5)	0.92 (0.69 - 1.22)	0.48 (0.35 - 0.66)^***^	0.26 (0.18 - 0.36)^***^
Any indicator of severity^1^								
No	168 (53.7)	1.00 (0.77 - 1.29)	0.91 (0.69 - 1.21)	0.79 (0.59 - 1.06)	411 (62.7)	1.32 (1.05 - 1.66)^*^	0.82 (0.63 - 1.06)	0.40 (0.31 - 0.53)^***^
Yes	137 (43.9)	0.54 (0.43 - 0.69)^***^	0.54 (0.42 - 0.70)^***^	0.46 (0.36 - 0.61)^***^	437 (59.2)	1.04 (0.85 - 1.27)	0.65 (0.52 - 0.81)^***^	0.31 (0.25 - 0.40)^***^
Any indicator of risk^2^								
No	205 (51.4)	0.84 (0.67 - 1.05)	0.81 (0.63-1.03)	0.70 (0.55 - 0.90)^**^	535 (62.9)	1.31 (1.07 - 1.60)^**^	0.79 (0.63 - 1.00)^*^	0.38 (0.30 - 0.49)^***^
Yes	100 (44.2)	0.56 (0.42 - 0.74)^***^	0.53 (0.39 - 0.73)^***^	0.46 (0.34 - 0.63)^***^	313 (57.6)	0.97 (0.77 - 1.23)	0.62 (0.48 - 0.80)^***^	0.31 (0.24 - 0.41)^***^

Eligible population includes non-linked non-SMI and linked SMI group

*p < 0.05, **p < 0.01, ***p < 0.001

^1^ Includes any of: ever had an inpatient stay, any record of being treated under the Mental Health Act, any record of difficulty managing their physical health, or any record of an Assertive Outreach/Crisis/A&E episode

^2^ Includes any of: recorded history of violence, recorded history of non-compliance, and any record of a forensic history

^a^ Adjusted for age (continuous), ethnicity, and borough-level deprivation

^b^ Additionally adjusted for mean annual number of primary consultations
